# Experimental studies of erythropoietin protection following traumatic brain injury in rats

**DOI:** 10.3892/etm.2012.723

**Published:** 2012-09-25

**Authors:** FENG XU, ZHENG-YUAN YU, LI DING, SHI-YING ZHENG

**Affiliations:** 1 Department of Emergency Surgery, The First Affiliated Hospital of Soochow University; 2 Department of Cardio-Thoracic Surgery, The First Affiliated Hospital of Soochow University, Suzhou, Jiangsu 215006, P.R. China

**Keywords:** erythropoietin, traumatic brain injury, apoptosis, monocyte chemotactic protein-1, CD68^+^ cells

## Abstract

This study aimed to explore the effect of erythropoietin (EPO) on brain tissue after traumatic brain injury in rats. Animals were divided into sham, control and EPO groups. The model was constructed using the improved Feeney’s free falling weight traumatic brain injury model. The brain water content and the number of the apoptotic monocyte chemotactic protein-1^+^ (MCP-1^+^) and CD68^+^ cells were monitored at 12, 48 and 120 h post-trauma. The water content was lower in the EPO group at each time point compared to the control group. The number of apoptotic MCP-1^+^ and CD68^+^ cells surrounding the traumatic brain injury lesion was less in the EPO group compared to these values in the control group. In conclusion, following traumatic brain injury, EPO significantly decreased the number of apoptotic cells, the expression of MCP-1, the infiltration of CD68^+^ cells as well as brain edema to protect the brain.

## Introduction

Craniocerebral injury is a common serious problem in neurosurgery, with extremely high mortality and disability rates. In craniocerebral injury, traumatic lesions show hemorrhagic necrosis and surrounding nerve cells exhibit apoptosis, when mediators of inflammation are released. Inflammatory cell infiltration and brain edema are actively involved in the pathophysiological mechanism, and the protection of the nerve cells still in a reversible state around traumatic lesions has become a focus of research in recent years.

Erythropoietin (EPO) has been shown to promote neuro-protection in hypoxic-ischemic cerebral insults, through the regulation of neurogenesis and preventing neuronal apoptosis (1–3). EPO, a vital compound of erythroid differentiation, is also involved in non-hematopoietic tissue-protective pathways, demonstrating antiapoptotic, anti-inflammatory, angiogenetic and neurotrophic properties (4). Hypoxia has been proven to increase EPO production in kidneys, the brain, the testis, the liver and the spleen (5–7). In the brain, EPO is highly expressed after a neuropathological insult (5). Astrocytes produce EPO after its hypoxia-induced upregulation, while neurons express EPO receptors (2,7). EPO has also been demonstrated to promote neuroprotection after systemic administration, even in severe cerebral ischemia (8), to enhance neurological recovery in traumatic brain and spinal cord injury (5,9–12), and to prevent the loss of autoregulation of cerebral blood flow (13). The neuroprotection and restructuring of cerebral tissue after a neuropathological trauma is likely to result in the use of EPO in clinical practice to limit neuronal damage (14).

This study was aimed to investigate the impact of EPO on nerve cell apoptosis in rats subsequent to experimental brain injury, the release of inflammatory mediators, inflammatory cell infiltration and brain edema.

## Materials and methods

### Experimental materials

The apoptosis detection kit, as well as the monocyte chemotactic protein-1 (MCP-1) monoclonal and CD68 polyclonal antibodies were purchased from Crystal US Biological (Downers Grove, MO, USA), the erythropoietin was purchased from Kirin Co., Ltd (Tokyo, Japan), while the head blow device was constructed in-house.

### Animal grouping

Ninety healthy male SD rats (provided by the Animal Center of the Soochow University School of Medicine), weighing 250–300 g, were randomized into a sham operation group, a control group and an EPO group (n=10/group). Five rats of each group were used to measure the number of apoptotic, MCP-1^+^ and CD68^+^ cells, while the remaining 5 were measured for brain water content.

### Production of head blow device

The improved Feeney’s method was employed (15) while following the free-falling principle to produce a head blow device, comprising a collision bar, a drop hammer, a peripheral casing and fixed devices. The head end of the collision bar had a diameter of 4.5 mm and a height of 3 mm, while the drop hammer weight was 20 g. The peripheral casing had a height of 30 cm, and the hit height could be randomly adjusted.

### Animal models

The improved method of Feeney *et al* (15) was employed to produce a free-falling model of brain contusion. The anesthetized rats were injected in the stomach using 1% pentobarbital at the proportion of 30 mg/kg, their heads were secured in a prone position and a sagittal scalp incision was made to expose the left parietal bone, and a hole of 2 mm was placed by a drill in front of the lambdoid suture and 2 mm to the left of the midline, the bone was expanded by a window of 5×5 mm; the impact force of the falling body was 20×30 g/cm that caused brain injury to the left hemisphere. After stopping the bleeding, bone wax was used to seal the bone window and the scalp was sutured. Except for the brain contusion impact, the same steps were repeated in the sham operation group. Having successfully established the animal model, the EPO group was injected with EPO 5,000 μ/kg, while the sham operation and the control groups were injected with a normal amount of saline. Subsequent to the operation the animals were caged individually. The heads of the animals in each group were removed to excise the brains 12, 48 and 120 h after the injury.

### Preparation of the tissue sections

Rats in each group were anesthetized with an overdose of 1% pentobarbital (40 mg/kg) at each time point. The brains were excised immediately after and secured for 30 min with 4% paraformaldehyde buffer. Taking the contusion as the center, four 5 μm-thick conventional paraffin sections were cut. Subsequently, H&E, TUNEL and MCP-1 and CD68 immunohistochemistry stainings were conducted, respectively.

### TUNEL staining

The slices were dewaxed by xylene, gradient alcohol hydration was conducted and digestion was carried out for 20 min with 20 μg/ml proteinase K, using 0.3% hydrogen peroxide and methanol to block endogenous peroxidase for 30 min. Then the sections were washed for 5 min (4°C) with 0.1% Triton X-100 and sodium citrate buffer liquid. Subsequently, 50 μl TUNEL reactive mixture was dripped onto the sections and they were incubated for 1 h at 37°C. After rinsing with phosphate-buffered saline (PBS), 50 μl converter POD was dripped onto the sections and was incubated for 30 min at 37°C. The sections were then washed with PBS and colored with 0.05% DAB for 15–20 min. The negative control operations were the same as the above, with the exception of the TUNEL reactive mixture. The apoptotic nuclei demonstrated brown staining. Five high-magnification views (×400) from around the contusion of each section were selected to count the number of positive cells in each view, and the average value was determined.

### Immunohistochemistry of MCP-1 and CD68

The paraffin-embedded brain tissue specimens were cut into 5 μm-thick sections. The ABC method was applied and the steps were as follows. i) Paraffin sections were prepared for brain tissue and dewaxing; ii) they were washed in PBS twice, and placed in 0.3% H_2_O_2_ methanol solution. The sections were kept at room temperature for 20 min, then washed 3 times in PBS solution to remove endogenous peroxidase activity. iii) The slices were placed in an incubator at 37°C with 0.1% trypsin for 20 min, then washed 3 times in PBS. iv) Subsequent to adding normal calf serum at a proportion of 1:10, the sections were kept at room temperature for 20 min. v) Subsequent to adding the first antibody at a working concentration, the sections were incubated at 37°C for 1 h, and washed 3 times in PBS solution. vi) After the addition of the second biotinylated antibody at a working concentration, the sections were incubated at 37°C for 1 h, and washed 3 times in PBS solution. vii) Subsequent to adding ABC complex, the sections were incubated at 37°C for 1 h, and washed 3 times in PBS solution. viii) AB color was developed to brownish red, and viiii) after a thorough washing in water, the sections were counterstained with hematoxylin. x) The sections were dehydrated, made transparent and mounted. PBS was used to replace the first antibody as a negative control. Cells staining with a granular brown-red color in the cytoplasm at a high magnification were considered positive cells. The number of positive cells were then counted (5 fields of view).

### Determination of brain water content

Brain hemisphere tissue samples were taken from rats in each group. Their wet weights was measured on electronic scales, at room temperature (20–25°C) and in 70–90% humidity. Subsequently, the samples were placed in an oven at 100±2°C for 24 h, and the dry weight was measured. The water content in the brain was calculated using the following formula: Wet weight - dry weight/wet weight x 100%.

### Statistical analysis

The data are presented using the SPSS statistical software, and the variable data were expressed as the means ± SD. The data were analyzed by ANOVA and detection measures.

## Results

### H&E staining

The trauma control group exhibited unstructured necrosis at different time points, showing edema of the hemorrhagic focus and peripheral cells and the broadening of tissue space. Numerous apoptotic neurons as well as the obvious infiltration of inflammatory cells were visible in the center of the trauma and in the peripheral edema. The center of the necrotic area and the peripheral edema in the EPO group was smaller compared to the control group, and was characterized by the alleviation of peripheral edema, a decreasing number of apoptotic neurons and the reduced infiltration of inflammatory cells. In the sham group only a small number of apoptotic cells were detected, while inflammatory cell infiltration was absent.

### Evaluation of apoptosis by TUNEL staining

**Figure 1:**
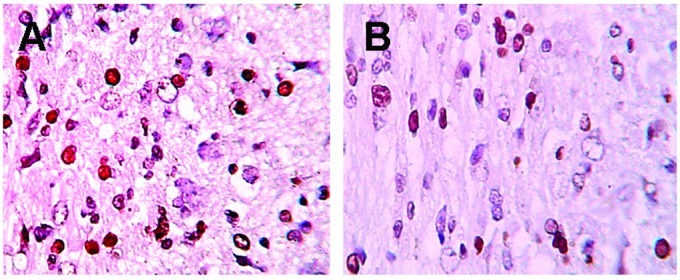
TUNEL staining showing apoptotic neurons 48 post-trauma in the (A) control and (B) EPO groups (×400) is shown.

In TUNEL staining (Fig. 1), more apoptotic neurons were detected in the control group at various time points. A clear decrease in apoptotic neurons (P<0.01) was detected in the EPO group compared to the control group, while only a few positive cells were detected in the sham group (Table I).

### MCP-1 staining

**Figure 2:**
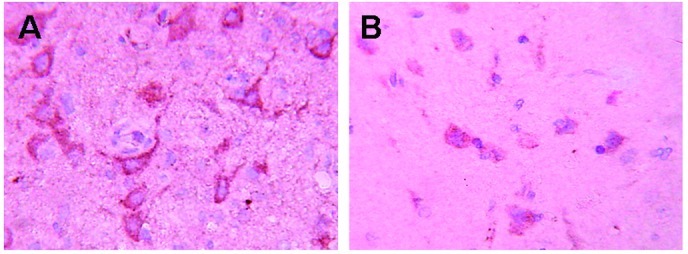
Immunohistochemical staining of MCP-1 in the (A) control and. (B) EPO groups (×400) is shown.

In the control group (Fig. 2), a large number of MCP-1^+^ cells were detected in the periphery of the trauma at different time points. that was most obvious after 48 h. In the EPO group the positive cells showed a clear decrease (P<0.01) at different time points, compared to the control group, while no positive cells were detected in the sham group (Table II).

### Infiltration of CD68^+^ cells

**Figure 3:**
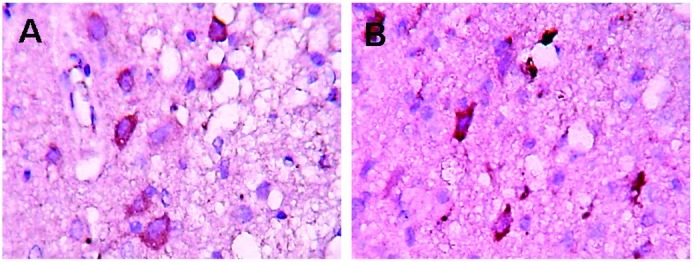
Infiltration of CD68^+^ cells in the (A) control and (B) EPO groups (×400) is shown.

In the control group, the infiltration of CD68^+^ cells was detected in the periphery of the trauma 12 h post-trauma. The largest number of positive cells were found at 120 h. The positive cells in the EPO group showed a clear decrease (P<0.05) compared to the control group (Fig. 3 and Table III).

### Water content of brain tissue

Compared to the sham group, an increase of the water content was observed in the brain tissue 12 h post-trauma, with the highest content at 48 h. Cerebral edema was noted to subside after 120 h. Compared to the control group, the water content in the brain tissue in the EPO group was the lowest (Table IV).

## Discussion

EPO is a glycoprotein hormone, with a molecular weight of 34 kDa. EPO existing in plasma consists of 165 amino acids, with a high glycosylation degree containing mainly sialic acid. It is located on human chromosome 7p22, including at least 5 introns. EPO exerts its effects by binding to its receptor EpoR, and is produced mainly by the kidneys. In recent years, numerous studies have shown that EPO and EpoR expression levels were detected in human brain tissue and confirmed that the degree of glycosylation in this type of EPO is different compared to the EPO in serum. The former contains less sialic acid, while having a stronger function. Astrocytes in the brain, neurons, microglia and endothelial cells are able to produce EPO. RT-PCR has demonstrated that mRNA of EPO and EpoR is widespread in the hippocampus and the cerebral cortex, while the production of EPO is correlated with blood, as well as the oxygen supply in brain tissue; when the brain lacks blood or oxygen, the generating of EPO increases, protecting neurons (16,17).

Research has shown that EPO has a strong effect on neuronal apoptosis, as well as on the inflammatory response. Siren *et al* (3) used an arterial occlusive model in rat brain and found that apoptotic neurons in the ischemic penumbral region in the experimental group (embolism and intravenous injecting EPO) were markedly less or even non-existent compared to the control group. Notwithstanding, the infarct size was significantly reduced after a 24-h occlusion of the brain artery. In pure or mixed medium neural cells, EPO 0.1–10.0 μ/ml blocked serum loss, as well as kainic acid exposure-induced apoptosis. In their study conducted on experimental spinal cord injury, Gorio *et al* (11) found that the sports function of the 5,000 U/kg EPO treatment group was significantly improved, while the contusion focus was reduced by 25%. The motor neurons in the central gray substance in the contusion area exhibited no TUNEL^+^ cells, and the infiltrate inflammatory cells were largely reduced, while the control group had a wide range of motor neuron injuries, TUNEL^+^ expression and visible inflammatory cell infiltrates. Agnello *et al* (18) found that in autoimmune myelitis, EPO significantly reduced monocyte/macrophage infiltration in areas of spinal cord inflammation, microglial cell activation, decreased the production of IL-6, while delaying the appearance of the TNF-α peak. In their studies, Chong *et al* (19) and Chen *et al* (20) found that endogenous EPO is not sufficient to maintain cell survival during acute injury, while EPO at a dose of 0.01-10 μ/ml for acute or chronic neuronal injury provides broad neural protection and subsequent microglial activation In this study, the EPO treatment group also showed an obvious inhibitory effect, as well as an inflammatory reaction, in the area around the injury. Cell apoptosis was significantly reduced, while MCP-1^+^ and CD68^+^ cells also decreased in number. In addition, this study also demonstrated that EPO has the potential to reduce the water content of brain tissue, indicating that EPO reduces traumatic brain edema. H&E staining also confirmed that EPO affected brain injury compared to the control group. The size of the injury area in the EPO group was markedly reduced, the number of degenerated and necrotic nerve cells and obvious infiltrating inflammatory cells was also decreased, whereas the brain edema was palliated. The protection of EPO on cell apoptosis after cerebral contusion involves the EpoR, although its specific anti-apoptotic mechanism is not clear. The research conducted on the mechanism of the production of erythrocytopoiesis by EPO shows that EPO acts on EpoR-activated tyrosine kinases JAK2 and JAK2 tyrosine phosphorylation of EpoR residues, signals molecules in activated cells, such as signal transducer and activator of transcription 5 (STAT5), phosphatidylinositol 3-kinase (PI3K), extracellular signal-regulated kinase 1,2 (ERKs), Ras protein/mitogen-activated protein kinase (Ras/MAPK) and nuclear transcription factor-κB (NF-κB). EPO also activates EpoR-JAK2-STAT5, EpoR-JAK2-PI3K, EpoR-JAK2-ERKs, EpoR-JAK2-Ras/MAPK, EpoR-JAK2-NF-κB and other pathways, while anti-apoptosis occurs through the upregulated protective gene transcription and protective protein expression. Digicaylioglu *et al* (21) found that pre-treatment with EPO significantly reduces neural cell apoptosis, while EpoR activation inhibits N-methyl-D-aspartate (NMDA)- and NO-induced apoptosis. The mechanism of the activation of JAK2 and JAK2 through EpoR, combined with the suppression of the cytoplasmic transcription factor-κB (NF-IκB) to phosphorylate IκBα tyrosine residues result in IκB degradation and NF-κB release. NF-κB migrates into the center of the nucleus to activate target genes, induce apoptosis and restrain the upregulation proteins XIAP and c-IAP2. Moreover, XIAP and c-IAP2 block the final path of activation of caspase in order to inhibit apoptosis. In addition, NF-κB migration increases the activity of glutathione, manganese, Cu and Zn-SOD to suppress neuronal apoptosis induced by the accumulation of superoxide anions, such as O_2_^−^ and ONOO. Chong *et al* (22) demonstrated that EPO has an obvious protective function on the neuronal damage-induced free radicals, such as NO, and suggested EPO to be involved through the activation of the extracellular signal-regulated kinase and protein kinase Akt1 or protein kinase B, 1. Active Akt1 activates Bad through phospho-serine 136 in a Bad protein, while the activated Bad induces the fusion of anti-apoptotic BCL-2/BCL-XL in order to inhibit neuronal apoptosis. Active Akt1 also restrains the depolarization of free radical-induced mitochondrial transmembrane potential, while the activation of caspases-8, -1 and -3 is known to be the last channel of apoptosis.

By regulating microglial activation and controlling cytokine release, EPO is highly involved in anti-inflammation. Due to the depolarization of the mitochondrial membrane potential and the release of cytochrome C, the injury to nerve cells induces the activation of caspases-8, -1 and -3. In particular, the activation of caspase-1 induces cell membrane phosphatidylserine exposure through the digestion of cytoskeletal protein, while the exposed phosphatidylserine participates in the activation and proliferation of microglial cells (23). In inflammatory responses of the central nervous system, the microglial cell is the first and most important component of inflammatory cells. Consequently, activated microglial cells are likely to upregulate large numbers of cell surface receptors, while releasing numerous pro-inflammatory factors, as well as toxic substances, such as TNF-α, IL-1β, NO, superoxide and fatty acid metabolism (24). Moreover, these pro-inflammatory factors and toxic substances are also likely to result in peripheral blood PMNs, monocytes/macrophages and lymphocytes, as well as local activated microglial cells infiltrating the area of the injury and around it. By releasing lysosomal enzymes, oxygen metabolism, inflammatory mediators and proinflammatory factors, activated microglial cells may worsen the original inflammation while inducing the accumulation of more inflammatory cells in the injury area, forming a vicious cycle that results in the expansion of the scope of injury. By inhibiting microglial activation and the release of its downstream inflammatory factors, EPO thereby restrains the local inflammatory response. In addition, EPO directly inhibits the pro-inflammatory factors and the activation of IL-6, TNF-α and MCP-1 to inhibit the inflammatory activity (3). Immediately after brain injury, MCP-1 is expressed mainly by astrocytes in the area around the injury, and afterwards mainly by infiltrating monocytes/macrophages and activated microglia (25). MCP-1 is the most important cell factor leading to the activation of brain microglia, peripheral blood monocytes/macrophages and lymphocyte infiltration in the area around the injury, whereas the activated microglia and other inflammatory cells produce large amounts of neurotoxic factors, leading to the degeneration and necrosis of nerve cells. By restraining the activity of MCP-1, EPO exhibits anti-inflammatory properties.

The experimental results showed that the water content in the cerebral hemisphere of the rats in the EPO treatment group at each time point was markedly reduced (P<0.01) compared to the control group, suggesting that EPO decreased traumatic brain edema. EPO functions by directly inhibiting apoptosis in microvascular endothelial cells to maintain the integrity of vascular endothelia. Chong *et al* (26) confirmed that EPO prevents hypoxia-induced vascular endothelial injury, while maintaining mitochondrial membrane stability by directly activating protein kinase B/Akt and inhibiting the cysteine protease caspases-8, -1 and -3 activity, in order to prevent the endothelial cell apoptosis in turn. In addition, EPO clearly inhibited local inflammatory reactions and reduced the local inflammatory vasoactive substances and cytotoxic factors that are potentially involved in the inhibition of brain edema. Martinez-Estrada *et al* (27) injected VEGF into the body of animals to produce an animal model with a blood-brain barrier opening and found that EPO markedly inhibited the damage caused by blood-brain barrier permeability maintaining vascular tight junctions between endothelial cells.

In conclusion, in this study, the protective effect of EPO on injured brain tissue was achieved through one or a series of ways, as described above, with a view to reduce cell apoptosis around the edema contusion, the inflammation response, and cerebral edema, while being involved in cerebral protection. In addition, EPO demonstrated neurotrophic factors, promoting the re-angiogenesis of blood vessels and accelerating the recovery of the imbalance of the self-regulation of cerebral blood flow. EPO has been widely used in clinical practice and has been proven to be a safe medication with little negative effect. Ultimately, with additional advances in research, EPO is likely to be used in clinical application as a safe and effective neuroprotective agent.

## Figures and Tables

**Table I:** Comparison of the apoptotic neurons in the sham, control and EPO groups.

Groups	No. of cases	12 h	48 h	120 h
Sham	5	1.56±0.68	1.40±0.80	1.68±0.82
Control	5	11.64±1.73	38.32±4.78	21.00±2.62
EPO	5	4.08±2.13^a^	24.40±2.28^a^	13.16±3.43^a^

a Means compared to the control group, P<0.01. EPO, erythropoietin.

**Table II:** Comparison of the MCP-1^+^ cells in the sham, control and EPO groups.

Groups	No. of cases	12 h	48 h	120 h
Sham	5	-	-	-
Control	5	14.00±2.12	33.20±2.71	20.80±2.24
EPO	5	4.32±1.67^a^	16.04±3.62^a^	10.04±0.90^a^

a Means compared to the control group, P<0.01. EPO, erythropoietin.

**Table III:** Comparison of the infiltration of CD68^+^ cells in the sham, control and EPO groups.

Groups	No. of cases	12 h	48 h	120 h
Sham	5	9.36±0.56	1.40±0.80	1.68±0.82
Control	5	18.68±1.96	26.08±3.17	39.12±3.70
EPO	5	12.60±2.75^a^	16.24±3.14^a^	27.12±4.78^a^

a Means compared to the control group, P<0.05. EPO, erythropoietin.

**Table IV:** Comparison of the water content in brain tissue in the sham, control and EPO groups.

Groups	No. of cases	12 h	48 h	120 h
Sham	5	77.02±0.74	77.08±1.23	77.49±0.30
Control	5	80.69±0.75	81.42±0.42	80.47±0.86
EPO	5	78.48±0.54^a^	79.31±0.85^a^	78.49±0.30^a^

a Means compared to the control group, P<0.01. EPO, erythropoietin.
